# Correlation between sarcopenia and cirrhosis: a meta-analysis

**DOI:** 10.3389/fnut.2023.1342100

**Published:** 2024-01-10

**Authors:** Yifan Cui, Mingming Zhang, Jing Guo, Jin Jin, Haijiao Wang, Xinran Wang

**Affiliations:** General Surgery Department, Xuanwu Hospital Capital Medical University, Beijing, China

**Keywords:** sarcopenia, cirrhosis, hepatic encephalopathy, survival rate, mortality

## Abstract

**Background:**

The relationship between sarcopenia and cirrhosis is unclear. In this research, our aim is to evaluate the prevalence of sarcopenia among individuals with liver cirrhosis and its correlation with survival and mortality risks.

**Methods:**

We conducted searches on PubMed, Web of Science, EMBASE, and Cochrane for English articles published up to July 10, 2023, and additionally manually searched the bibliography of relevant articles. We incorporated research on sarcopenia in patients with cirrhosis to examine the connection between sarcopenia and the likelihood of survival and mortality. Statistical analyses were carried out utilizing the Stata version 15.1 software. Depending on the heterogeneity of the results, we employed either fixed-effects models or random-effects models for data synthesis. To assess publication bias, we employed funnel plots and conducted Egger’s test.

**Results:**

We included 40 studies involving 8,945 patients with cirrhosis. The overall prevalence of cirrhosis was 41% (95% CI 34%–48%). Male patients and those with liver cirrhosis and hepatic encephalopathy had a higher prevalence of sarcopenia (44% for male patients and 48% for hepatic encephalopathy patients). Sarcopenia emerged as a risk factor for both survival (HR = 2.57, 95% CI 2.02–3.27, *p* < 0.001) and mortality (HR = 2.13, 95% CI 1.86–2.44, *p* < 0.001) in patients with cirrhosis. Subgroup analyses consistently yielded the same results for study sites, whether HCC patients were excluded from the cohort, whether patients were from the liver transplant cohort or had undergone tips surgery, the definition of sarcopenia (L3-SMI or other methods), and the diagnostic criteria used by patients. The presence of sarcopenia was also a significant risk factor for hepatic encephalopathy [HR = 2.27, 95% CI (1.76–2.94), *p* < 0.001].

**Conclusion:**

This systematic review and meta-analysis reveal that patients with cirrhosis have a prevalence of sarcopenia of 41% and is associated with survival rate and mortality rate. Therefore, we should attach importance to the screening of sarcopenia in patients with cirrhosis, early detection of susceptible populations, and appropriate measures to reduce the occurrence and adverse outcomes.

**Systematic review registration:**https://www.crd.york.ac.uk/PROSPERO/#recordDetails.

## 1 Introduction

Cirrhosis is the advanced stage of chronic liver diseases and is mainly attributed to hepatitis B or C virus infection, alcohol consumption, non-alcoholic fatty liver disease, and autoimmune diseases (1). This disease has resulted in more than 1.3 million deaths, making it one of the leading global causes of mortality (2). With disease progression, many complications follow, including ascites, variceal bleeding, and hepatic encephalopathy (HE), which considerably affect the prognosis of patients with cirrhosis (3). HE is defined as a spectrum of nonspecific neurological or psychiatric abnormalities ranging from subclinical alterations to coma. It is typically induced by liver failure and/or portal vein-systemic shunting. As one of the primary complications of late-stage cirrhosis, HE has an incidence rate of about 20% to 80% (4). In patients with cirrhosis, elevated ammonia concentrations, brain edema, and increased intracranial pressure can contribute to varying degrees of HE (5, 6). HE is associated with a poor prognosis, often necessitating frequent hospitalization, imposing socio-economic and psychological burdens on patients and their families, and ultimately reducing the overall survival rate (7). Therefore, it is pressingly urgent to find more risk factors for cirrhosis.

Sarcopenia is a prevalent concern in individuals diagnosed with liver cirrhosis (8). Characterized by the decline in muscle mass, strength, and physical performance (9), sarcopenia has been detected in 14% to 55% of cirrhosis patients, prompting growing interest among researchers (10). The liver holds a central position in nutrient metabolism, and the diminishment of liver functional reserves can result in a range of complications, including malnutrition and the development of sarcopenia (11). Previous studies have revealed that sarcopenia in cirrhosis patients may be attributed to liver metabolic dysfunction, reduced appetite, increased muscle autophagy, elevated serum myostatin levels, catabolic effects of systemic inflammation induced by intestinal bacterial translocation, and low testosterone levels (10). Sarcopenia in cirrhosis patients is associated with a grim prognosis (12), such as reduced quality of life, elevated hepatic venous pressure gradients, complications related to portal hypertension (such as ascites and upper gastrointestinal varices), infections (including urinary tract infections and spontaneous peritonitis) and HE (10). Reduced extrahepatic ammonia clearance in patients with sarcopenia may contribute to HE to some extent (13).

It has been demonstrated that sarcopenia affects both hepatic encephalopathy and mortality in cirrhosis patients (13), and the decline in muscle mass in cirrhosis patients for more than a year also carries unfavorable prognostic implications (14). A meta-analysis of 22 studies has confirmed that sarcopenia is an independent predictor of increased mortality in cirrhosis patients (15). The progressive and systemic loss of skeletal muscle mass and strength in patients with sarcopenia also indicates, to some extent, the poor prognosis of LC patients (16). Therefore, we have embarked on an up-to-date and more exhaustive meta-analysis to comprehensively investigate the consequences of sarcopenia in individuals with cirrhosis. We aim to appraise the survival rates and mortality rates in cirrhotic patients with sarcopenia. The secondary objectives include appraising the prevalence of sarcopenia in cirrhotic patients and examining the impact of sarcopenia on LC and HE.

## 2 Methods

This meta-analysis followed the updated PRISMA (2020) and MOOSE guidelines (17–19), and the study protocol was registered with PROSPERO (CRD42023458935).

### 2.1 Search strategy

We conducted a comprehensive search on PubMed, Embase, Cochrane, and Web of Science, spanning from the inception of these databases to July 10, 2023, with the language limited English. The combination of medical subject heading (MeSH) terms and their free-form words was used as the search strategy, such as sarcopenia [MeSH Terms] AND liver cirrhosis [MeSH Terms] OR hepatic encephalopathy [MeSH Terms]. Supplementary Table S1 provides detailed search strategies for all the included databases. We restricted our search to studies involving human subjects to maintain relevance. In our pursuit of thoroughness, we expanded our search efforts to include conference abstracts from major events such as the 2019–2020 American Association for the Study of Liver Diseases (AASLD), European Association for the Study of the Liver (EASL), Asia-Pacific Association for the Study of the Liver (APASL), Digestive Disease Week (DDW), and Asian Pacific Digestive Week (APDW) in the hopes of identifying additional research. Lastly, we conducted a manual examination of the reference lists of the studies included in our analysis and relevant systematic reviews and meta-analyses to identify any potential studies that may have been missed through our initial searches.

### 2.2 Inclusion and exclusion criteria

The research explored the association between sarcopenia and liver cirrhosis or liver cirrhosis accompanied by hepatic encephalopathy.

Study eligibility criteria were based on the following PICO format: P (population): people with sarcopenia and cirrhosis (patients with or without hepatic encephalopathy were recorded separately); I (intervention/predictors): NA; C (comparator): people without sarcopenia; O (outcome): correlation between sarcopenia and cirrhosis (i.e., prevalence of sarcopenia, risk of survival and death, impact of myasthenia gravis and hepatic encephalopathy); S (study design): Observational studies (i.e., longitudinal, cross-sectional, and case control studies).

The studies were excluded for the following reasons: (1) comments, editorials, letters, posters, case reports, reviews, meta-analyses, conference abstracts, guidelines, and animal experiments; (2) no clear diagnostic criteria for cirrhosis and/or sarcopenia; (3) without enough data or unavailability of full-texts; (4) not published in English.

### 2.3 Literature screening

The titles and abstracts were screened independently using a pre-planned list of inclusion and exclusion criteria. Information such as varying criteria for defining sarcopenia, the general health status of patients, country of origin, recruitment background (hospital or nursing home), or research environment (cohort or cross-section) was not excluded. Following the inclusion criteria, we included studies that involved the population diagnosed with liver cirrhosis (LC) and provided some original data related to sarcopenia (prevalence rate, survival rate, mortality rate, etc.). Further meta-analyses were performed with patients stratified by the presence or absence of hepatocellular carcinoma as a covariate.

### 2.4 Quality evaluation

The quality of the included studies was assessed using the modified Newcastle–Ottawa scale (20). The scale consists of three sections with eight entries, which are as follows: (1) representativeness of the exposed cohort; (2) selection of the non-exposed cohort; (3) identification of exposure; (4) demonstration that no results of interest were found at the start of the study; (5) comparability of the cohorts based on design or analysis results controlled for confounders; (6) evaluation of results; (7) follow-up of sufficient duration to obtain results; and (8) appropriateness of follow-up to the population. The total score is 9 stars. Each study was independently assessed by two authors, and studies with NOS scores ≥6 were considered of high quality. Any disputed articles were referred to a third researcher, XW, for discussion and resolution of any disagreements.

### 2.5 Data extraction

We compiled data from each of the included studies utilizing a standardized table. The subsequent details were independently extracted by two assessors, YC and MZ: the primary author’s name, publication year, study design, research location, the origin of the cirrhosis cohort (transplant waiting list or general population), the definition of sarcopenia, the methodology employed for muscle mass measurement, the number of participants, patient demographics, and clinical characteristics, including age, gender, cirrhosis etiology, hepatocellular carcinoma (HCC) presence, and pertinent outcome measures such as sarcopenia incidence, survival rate, and mortality in cirrhosis patients. When the required data were not readily accessible, we made contact with the authors to secure the essential study information.

### 2.6 Statistical analysis

We conducted the statistical analysis using Stata version 15.1. The forest plot illustrates the overall effect of the analysis. Heterogeneity among the studies was assessed using *I*^2^. In general, *I*^2^ ≥ 50% indicated significant heterogeneity among the studies, leading us to adopt a random-effects model. We analyzed the source of heterogeneity through sensitivity analysis (one-by-one exclusion method). Additionally, pre-planned subgroup analyses were performed based on gender, age, etiology of cirrhosis, and study site. We employed meta-regression to determine the effects of sample size, mean age of participants, proportion of males, proportion of patients with alcohol-related liver disease, viral hepatitis, and the presence or absence of hepatocellular carcinoma (HCC) on the adjusted pooled hazard ratios (HR) for survival rate and mortality. Conversely, *I*^2^ < 50% was considered indicative of small heterogeneity across studies. We used funnel plots and Egger’s tests (21) to examine the possibility of publication bias. A *p*-value <0.05 (two-tailed test) was considered statistically significant.

## 3 Results

### 3.1 Literature screening process and results

**Figure 1:**
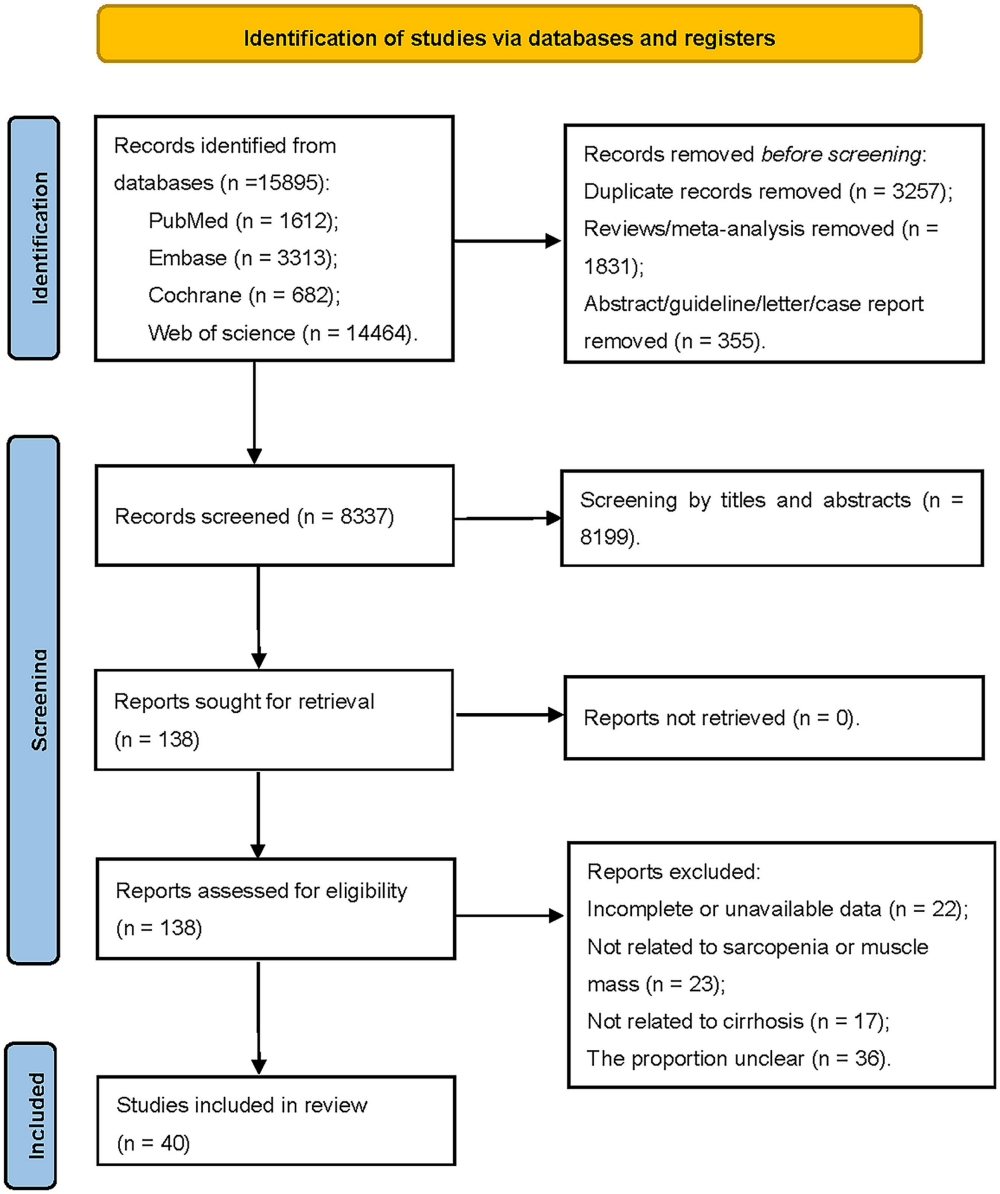
Literature screening flow chart.

Out of the 15,895 articles initially identified, we excluded 3,257 duplications and 4,339 articles classified as reviews, guidelines, letters, and animal experiments. An additional 8,199 papers were excluded based on a preliminary review of titles and abstracts. After carefully reviewing the full texts of the remaining 138 articles, we included 40 cohort studies with data from 8,945 patients (Figure 1).

### 3.2 Basic characteristics of the included literature

Table 1 summarizes the characteristics of all the studies encompassed in this meta-analysis. Out of the 40 studies, 18 were conducted in Asian regions (14, 22–24, 26, 29, 33, 35, 37, 38, 40, 44, 45, 48, 50, 53, 55, 59), and the remaining 22 studies were from non-Asian regions (13, 25, 27, 28, 30–32, 34, 36, 39, 41–43, 46, 47, 49, 51, 52, 54, 56–58). Among these 40 studies, 37 were cohort studies, 2 were cross-sectional studies (43, 57), and 1 was a case-control study (35). Specifically, 19 were retrospective cohort studies, and 18 were prospective cohort studies (13, 14, 27, 29, 30, 32, 33, 38–41, 49–57). The sample sizes in the included studies varied, ranging from 52 to 675 participants. In these studies, the mean age of patients ranged from 51 to 73 years. Notably, 24 studies (13, 14, 22–24, 27–29, 31, 33–35, 37, 38, 42, 44, 45, 48, 55, 57, 59) excluded all patients with hepatocellular carcinomas (HCC), while in 10 studies (28, 31, 37, 39, 46, 47, 49, 54, 55, 57), a proportion of patients ranging from 19 to 100% had HCC. Additionally, 8 studies (26, 39, 43, 46, 52, 54) neither explicitly excluded HCC patients nor reported the prevalence of HCC.

**Table 1:** Basic characteristics of included studies.

Author (year)	Study design	Country	Source of the sample	Definition of sarcopenia	Measure muscle mass	Sample size	Age (years)	Male (%)	HCC	ALD *N* (%)	Viral hepatitis *N* (%)
Yin et al. 2023 (22)	Cohort	China	Patients underwent the TIPS	Others	CT scan/L3 TPMT	108	53.0 ± 10.8	78	N	N	74 (69)
Saeki et al. 2023 (23)	Cohort	Japan	General patients	JSH	BIA/SMI	104	72.8 ± 3.2	56	N	N	66 (64)
Saeki et al. 2021 (23)	Cohort	Japan	General patients	JSH	BIA/SMI	126	70.2 ± 3.5	61	N	47 (37)	49 (39)
Hanai et al. 2023 (24)	Cohort	Japan	General patients	JSH	CT scan/L3 SMI	239	67.9 ± 3.2	52	N	60 (25)	85 (36)
Dajti et al. 2023 (25)	Cohort	Italy	General patients	EASL	CT scan/L3 SMI	209	64.2 ± 3.5	73	40 (19)	N	121 (58)
Nardelli et al. 2022 (13)	Cohort	Italy	General patients	EASL	CT scan/L3 SMI	114	58	68	N	9 (8)	7 (6)
Kumar et al. 2022 (26)	Cohort	India	General patients	Others	CT scan L3 PMI	74	51	96	Y	7 (10)	10 (14)
Kim et al. 2022 (14)	Cohort	Korea	General patients	AASLD	CT scan/L3 SMI	595	55.4 ± 7.9	64	N	99 (21)	319 (54)
Iacob et al. 2022 (27)	Cohort	Romania	General patients	EWGSOP2	CT scan/L3 SMI	71	54.5 ± 12.6	68	N	23 (32)	42 (59)
Hentschel et al. 2022 (28)	Cohort	Germany	General patients	FLEXIT	CT scan/L3 PMI	65	60.4 ± 12.8	65	N	30 (46)	3 (5)
Zeng et al. 2021 (29)	Cohort	China	General patients	Others	CT scan/L3 SMI	480	54	60	N	60 (20)	153 (32)
Topan et al. 2021 (30)	Cohort	Romania	General patients	EWGSOP2	CT scan/L3 SMI	201	61.7 ± 9.5	63	65 (32)	111 (55)	68 (34)
Paternostro et al. 2021 (31)	Cohort	Austria	Patients underwent HVPG-measurement	Others	CT scan or MRI/L3 TPMT	203	55 ± 11	68	N	110 (54)	50 (25)
Miarka et al. 2021 (32)	Cohort	Poland	General patients	EWGSOP	CT scan/L3 SMI	98	55 ± 8	77	26 (27)	50 (51)	37 (38)
Kikuchi et al. 2021 (33)	Cohort	Japan	General patients	JSH	CT scan/L3 SMI	300	69.1 ± 11.3	58	N	74 (27)	152 (51)
Welch et al. 2020 (34)	Cohort	Ohio	General patients	Others	CT scan/L3 SMA	83	52.7 ± 9.5	71	N	N	N
Tateyama et al. 2020 (35)	Case control	Japan	General patients	JSH	CT scan/L3 SMI	99	69.5 ± 9.6	61	N	39 (39)	70 (71)
Mauro et al. 2020 (36)	Cohort	Argentina	Listed for LT (before LT)	EWGSOP2	CT scan or MRI/L3 SMI	180	58.9 ± 2.2	60	N	50 (28)	47 (26)
Feng et al. 2020 (37)	Cohort	China	General patients	EWGSOP2	CT scan/L3 SMI	492	51.1 ± 2.7	74	N	60 (12)	333 (68)
Sung et al. 2019 (38)	Cohort	Japan	General patients	JSH	CT scan/L3 SMI	166	68.0 ± 8.2	58	N	50 (30)	85 (51)
Praktiknjo et al. 2019 (39)	Cohort	Germany	Patients underwent the TIPS	Others	CT scan/ umbilicus-TPMT	186	55.5 ± 11.4	59	Y	129 (77)	24 (13)
Ando et al. 2019 (40)	Cohort	Japan	General patients	JSH	CT scan/L3 SMI	88	67.3 ± 9.8	56	45 (51)	21 (24)	39 (44)
van Vugt et al. 2018 (41)	Cohort	Netherlands	Listed for LT (before LT)	Others	CT scan/L3 SMI	585	56.0 ± 2.3	69	193 (33)	91 (16)	52 (9)
Praktiknjo et al. 2018 (42)	Cohort	Germany	Patients underwent the tips	Others	MRI/FFMA	116	57.9 ± 12.0	59	N	73 (63)	18 (16)
Moctezuma-Velazquez et al. 2018 (43)	Cross-sectional	Canada	General patients	Others	CT scan/L3 SMI	210	57 ± 1	61	Y	54 (26)	74 (35)
Kang et al. 2018 (44)	Cohort	Korea	General patients	Others	CT scan/L3 SMI	452	51.8 ± 8.8	84	N	313 (69)	120 (27)
Jeong et al. 2018 (45)	Cohort	Korea	General patients	Others	CT scan/L3 SMA	131	53.7 ± 9.6	72	N	91 (70)	31 (24)
Bhanji et al. 2018 (46)	Cohort	Canada	General patients	Others	CT scan/L3 SMI	675	57 ± 1	67	Y	152 (22)	267 (40)
Begini et al. 2017 (47)	Cohort	Italy	General patients	Others	CT scan/L3 SMI	92	70.1 ± 11.3	71	92 (100)	22 (24)	51 (42)
Hanai et al. 2016 (48)	Cohort	Japan	General patients	Others	CT scan/L3 SMI	149	64.4 ± 11.4	55	N	34 (23)	89 (60)
Montano-Loza et al. 2015 (49)	Cohort	Canada	General patients	Others	CT scan/L3 SMI	669	57 ± 0.5	68	289 (43)	153 (23)	308 (46)
Hanai et al. 2015 (50)	Cohort	Japan	General patients	Others	CT scan/L3 SMI	130	65.4 ± 12.2	58	N	29 (22)	79 (61)
Tsien et al. 2014 (51)	Cohort	America	Listed for LT (before LT)	Others	CT scan	53	56.9 ± 7.5	77	34 (64)	12 (23)	34 (64)
Montano Loza et al. 2021 (52)	Cohort	Canada	Listed for LT (before LT)	Others	CT scan/L3 SMI	112	54 ± 1	70	Y	43 (38)	34 (30)
Anand et al. 2012 (53)	Cohort	India	General patients	Others	CT scan/L3 SMI	180	42.9 ± 9.9	79	N	47 (26)	N
Santos et al. 2019 (54)	Cohort	Brazil	General patients	ESPEN	DXA/AMMI	261	57.0 ± 1.9	62	Y	N	N
Murata et al. 2022 (55)	Cohort	Japan	General patients	JSH	CT scan/L3 SMI	151	70 ± 10	63	N	37 (25)	88 (58)
Salman et al. 2020 (56)	Cohort	Egypt	General patients	Others	CT scan/L3 SMI	52	53.9 ± 5.0	73	52 (100)	N	28 (54)
Ebadi et al. 2020 (57)	Cross-sectional	Canada	General patients	Others	CT scan/L3 SMI	603	57 ± 0.4	68	N	73 (18)	207 (34)
Kappus et al. 2020 (58)	Cohort	America	Listed for LT (before LT)	Others	CT scan/L3 SMI	355	54.7 ± 10.8	65	95 (27)	14 (4)	209 (59)

JSH, Japan Society of Hepatology; EASL, European Association for the Study of the Liver; ESPEN, European Society of Clinical Nutrition and Metabolism; AASLD, American Association for the Study of Liver Diseases; FLEXIT, Each center is a member of the Fitness, Life Enhancement, and Exercise in Transplant; LT, liver transplant; HCC, hepatocellular carcinoma; EWGSOP2, 2019 European Working Group on Sarcopenia in Older Adults; EWGSOP, 2010 European Working Group on Sarcopenia in Older Adults; AWGS, Asian Working Group for Sarcopenia; BIA, bioimpedance analysis skeletal; SMD, muscle radiodensity; FFMA, fat-free muscle area; L3 SMA, skeletal muscle area at the L3 vertebra; SMI, muscle mass index at the L3 vertebra; L3-PMI, the psoas muscle index at the L3 vertebra; L3 TPMT, transversal psoas muscle thickness at the L3 vertebra; AMMI, appendicular muscle mass index; CT, computed tomography; L3, The third lumbar vertebra; N, There were no such patients in the study population; Y, There are such patients in the study population; TIPS, Transjugular intrahepatic portosystemic shunt.

### 3.3 Quality evaluation

All studies were rated high quality with NOS scores ≥6. Among them, there were 5 studies (26, 28, 35, 55, 56) with 6 scores, 20 studies (13, 23, 24, 27, 32, 34, 36, 37, 39, 40, 43, 45, 47, 48, 51, 53, 54, 57–59) with 7 scores, and 15 studies (14, 22, 23, 29–31, 33, 38, 41, 42, 44, 46, 49, 50, 52) with 8 scores.

### 3.4 Meta-analysis

#### 3.4.1 Association between sarcopenia and cirrhosis

**Figure 2:**
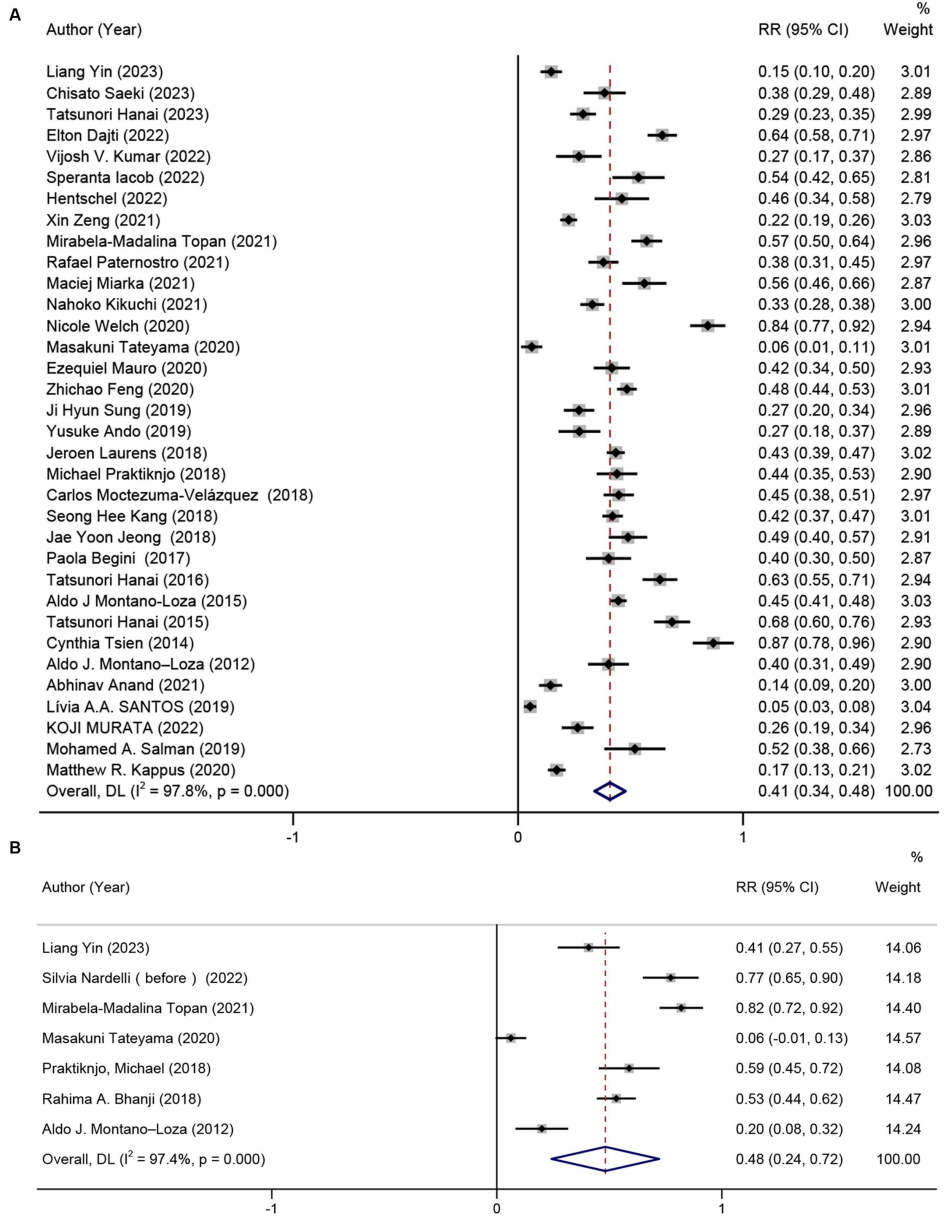
Prevalence of sarcopenia in patients with **(A)** cirrhosis, and **(B)** hepatic encephalopathy due to cirrhosis.

Out of the 40 studies included, 34 studies (22–38, 40–45, 47–56, 58) with a total of 7,024 participants provided data on prevalence of sarcopenia, resulting in a pooled prevalence of 41% (RR = 41, 95% CI: 34%–48%, *p* < 0.001). Due to significant heterogeneity between studies (*I*^2^ = 97.8%), as depicted in Figure 2A, subgroup analyses were conducted based on gender, the definition of sarcopenia, diagnostic criteria, and etiology of cirrhosis. The results revealed variations in prevalence between these subgroups. In particular, male patients exhibited a greater overall prevalence of sarcopenia in contrast to female patients. Patients diagnosed with sarcopenia according to the EWSOP2 criteria had a higher prevalence compared to those using other criteria (RR = 50, 95% CI: 44%–56%, *p* < 0.001). Furthermore, there was a higher prevalence of sarcopenia among patients in the liver transplant cohort compared to other patients (RR = 46, 95% CI: 17%–76%, *p* < 0.001). Notably, the prevalence of sarcopenia, as defined by the skeletal muscle area at the third lumbar spine (L3-SMA), was higher in the European population (RR = 67, 95% CI: 32%–100%, *p* < 0.001) compared to the Asian population (Europe, RR = 59, 95% CI: 38%–80%, *p* < 0.001; Asia, RR = 34, 95% CI: 25%–43%, *p* < 0.001). Surprisingly, the prevalence of patients with or without hepatocellular carcinoma was nearly the same (HCC, RR = 38, 95% CI: 26%–51%, *p* < 0.001; non-HCC, RR = 39, 95% CI: 31%–47%, *p* < 0.001). Furthermore, subgroup analyses indicated that factors such as gender, criteria for defining sarcopenia, methods of measuring muscle mass, patient origin, patient’s country, and the inclusion of HCC patients in the cohort were not sources of heterogeneity (Table 2). The result of Egger’s test indicated the presence of publication bias (*p* = 0.001).

**Table 2:** Subgroup analysis of the correlation between depression and Internet addiction based on gender, definition of sarcopenia, measure muscle mass, HCC and country.

Subgroup	RR	95% CI	*p*	*I* ^2^
**Gender**				
Men	44%	38%–51%	<0.001	95.2%
Women	35%	27%–44%	<0.001	95.0%
**Definition of sarcopenia**				
EWSOP2	50%	46%–53%	<0.001	67.5%
JSH	24%	21–26%	<0.001	92.6%
Others	37%	35%–38%	<0.001	97.5%
**Source of sample**				
General patients	41%	33%–49%	<0.001	97.9%
Patients underwent the TIPS	29%	0%–58%	<0.001	96.8%
Listed for LT (before LT)	46%	17%–76%	<0.001	98.5%
**Measure muscle mass**				
L3 SMA	67%	32%–100%	<0.001	97.2%
L3 SMI	40%	33%–46%	<0.001	96.6%
L3 PMI	36%	18%–55%	<0.001	82.3%
L3 TPMT	26%	4%–49%	<0.001	96.7%
**HCC**				
Y	38%	26%–51%	<0.001	98.3%
N	39%	31%–47%	<0.001	97.2%
**Country**				
Asia	34%	25%–43%	<0.001	97.8%
Europe	59%	38%–80%	<0.001	94.4%
North America	45%	36%–54%	<0.001	95.8%

EWGSOP2, 2019 European Working Group on Sarcopenia in Older Adults; JSH, Japan Society of Hepatology; EASL, European Association for the Study of the Liver; Y, There are such patients in the cohort, N, There were no such patients in the cohort; LT, liver transplant; TIPS, Transjugular intrahepatic portosystemic shunt; L3 SMA, skeletal muscle area at the L3 vertebra; SMI, muscle mass index at the L3 vertebra; L3-PMI, the psoas muscle index at the L3 vertebra; L3 TPMT, transversal psoas muscle thickness at the L3 vertebra; N, There were no such patients in the study population; Y, There are such patients in the study population.

#### 3.4.2 Association between sarcopenia and risk of hepatic encephalopathy in patients with cirrhosis

The prevalence of sarcopenia was reported in seven studies (13, 22, 30, 35, 42, 46, 52) (*n* = 426). The results unraveled that the pooled prevalence of sarcopenia was 48% (95% CI: 27%–72%, *p* < 0.001) in patients with cirrhosis and hepatic encephalopathy (Figure 2B). The presence of hepatic encephalopathy increased the prevalence of sarcopenia. However, there was significant heterogeneity among the studies (*I*^2^ = 97.6%). Egger’s test showed no significant publication bias (*p* = 0.217).

**Figure 3:**
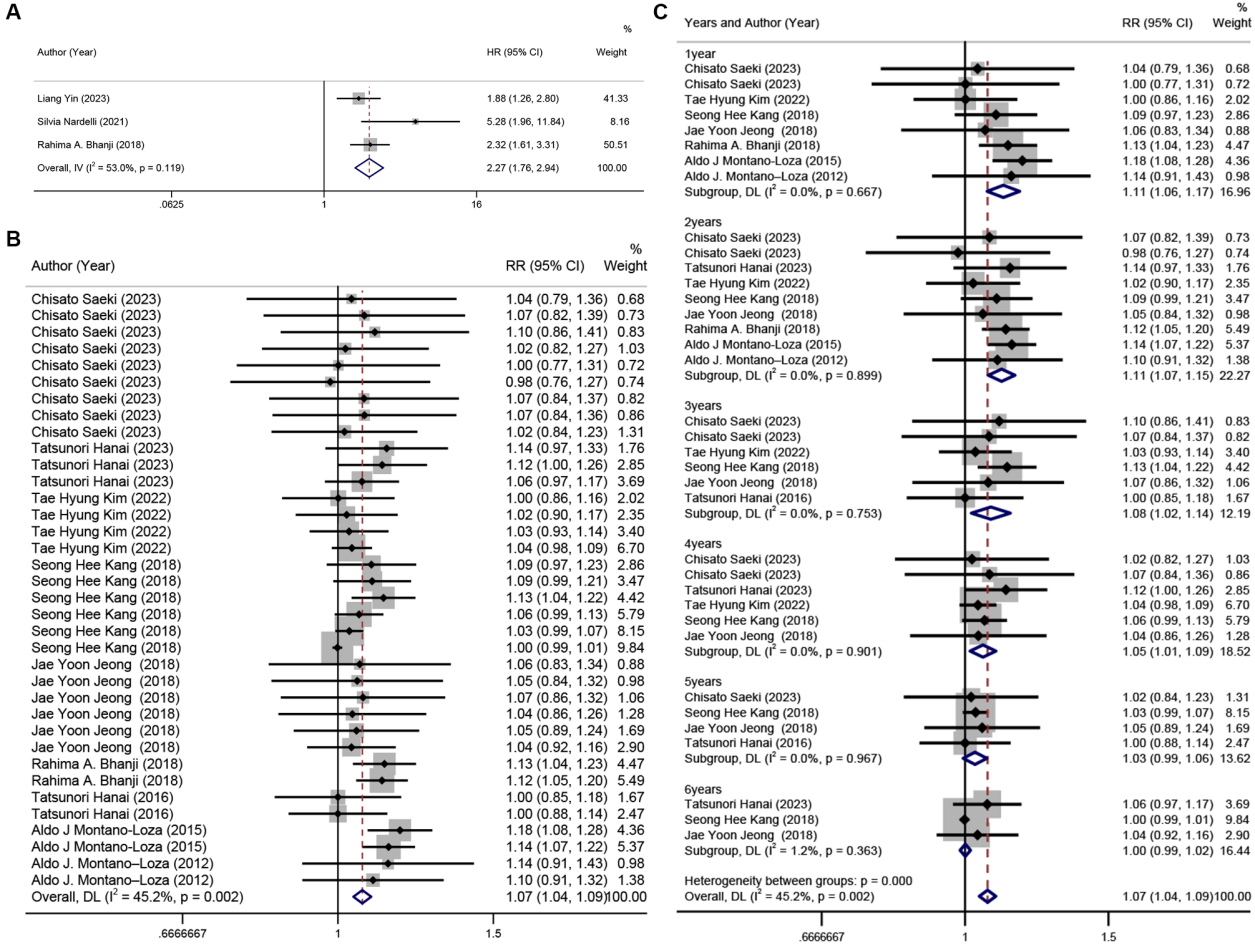
Forest plot. **(A)** univariate analysis of the incidence of hepatic encephalopathy in patients with sarcopenia and cirrhosis, **(B)** 6 years cumulative survival rate in patients with or without sarcopenia, **(C)** annual survival rate subgroups in patients with and without sarcopenia.

The HR results from the other three studies (13, 22, 46) were combined, revealing that sarcopenia was a risk factor for hepatic encephalopathy (HR = 2.27, 95% CI: 1.76–2.94, *p* < 0.001). Slightly greater heterogeneity was detected between the studies (*I*^2^ = 53%) (Figure 3A). The result of Egger’s test yielded a *p*-value of 0.323.

#### 3.4.3 Impact of sarcopenia on the cumulative survival rate among individuals with liver cirrhosis

Ten studies (14, 23, 24, 44–46, 48, 49, 52, 59) (*n* = 1,218) were included in this analysis. The results revealed that sarcopenia in patients with cirrhosis was a risk factor for a 6-year survival rate (RR = 1.07, 95% CI: 1.04–1.09, *p* < 0.001), with low heterogeneity between studies (*I*^2^ = 45.2%) (Figure 3B). However, based on the result of Egger’s test (*p* < 0.001), there was evidence of publication bias among the studies. Subgroup analysis by time (years) showed that sarcopenia had a more pronounced impact on survival rates in the first 4 years, which decreased with time (Figure 3C).

**Figure 4:**
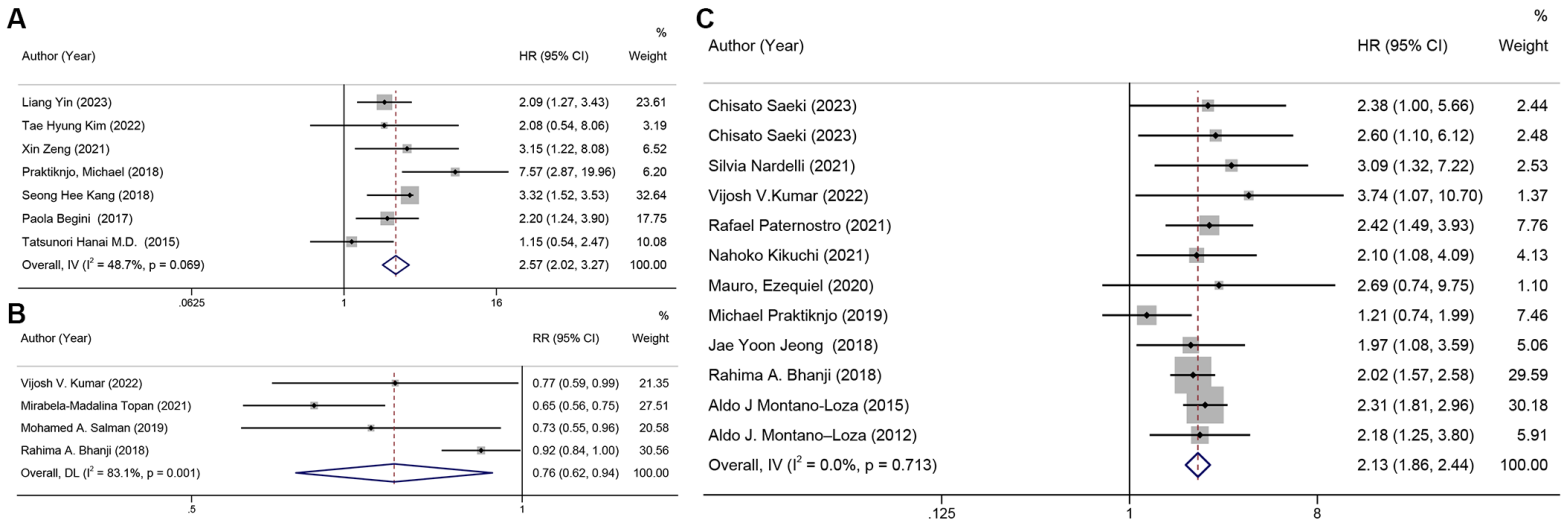
Forest plot. **(A)** univariate analysis of sarcopenia and survival rate, **(B)** mortality in patients with sarcopenia and cirrhosis, **(C)** univariate analysis of mortality in patients with sarcopenia and cirrhosis.

By analyzing the HR for cirrhosis survival and sarcopenia in seven of these studies (14, 22, 29, 42, 44, 47, 50) (*n* = 1,973), we found that sarcopenia was a barrier to survival (HR = 2.57, 95% CI: 2.02–3.27), with relatively low heterogeneity between studies (*I*^2^ = 48.7%) (Figure 4A).

When excluding the cohort that included HCC patients, a separate analysis of patients without HCC revealed a pooled HR of 2.44 (95% CI: 1.85–3.22). Furthermore, sarcopenia was associated with an over 2-fold reduction in survival rates in patients with liver cirrhosis, regardless of the study site, whether HCC patients were excluded from the cohort, whether patients underwent liver transplant or TIPS surgery, whether sarcopenia was defined by L3-SMI or other methods, and diagnostic criteria (Supplementary Figures S1–S3). The funnel plot is shown in Supplementary Figure S4.

#### 3.4.4 Correlation between sarcopenia and mortality in patients with cirrhosis

The mortality in patients with cirrhosis across four studies (26, 30, 46, 56) was meta-analyzed, and the pooled mortality rate was 76% (95% CI: 62%–94%, *p* = 0.011). There was a high likelihood of heterogeneity between the studies (*I*^2^ = 80.7%) (Figure 4B). In the sensitivity analysis, we excluded one study (46) after careful examination, and the pooled corrected mortality risk ratio (RR) was similar to that of the main analysis, at 68% (95% CI: 61%–77%, *p* < 0.001). Heterogeneity was very low (*I*^2^ = 0%). The result of Egger’s test was *p* = 0.464.

In a univariate analysis of data from 12 studies (13, 23, 26, 31, 33, 36, 39, 45, 46, 49, 52, 59) (*n* = 1,781), it was found that sarcopenia was a risk factor for mortality with a combined unadjusted hazard ratio (HR) of 2.13 (95% CI: 1.86–2.44, *p* < 0.001). The heterogeneity between studies was very low (*I*^2^ = 0%) (Figure 4C), and Egger’s test revealed no publication bias in the studies and that the combined HR was reliable (*p* = 0.959). The funnel plot is shown in Supplementary Figure S5.

In all subgroups of the mortality analysis, including country, definition of sarcopenia, presence of HCC, and source of the sample, sarcopenia consistently emerged as a risk factor for mortality. Regardless of the patient’s country and region, the definition standard of sarcopenia, and the method of measuring muscle mass, whether the cohort included ALD patients or excluded HCC patients, the HR for mortality in sarcopenia patients was consistently around 2 times higher (Supplementary Figures S6–S9).

## 4 Discussion

In this analysis of 40 studies involving 8,945 patients with cirrhosis, the prevalence of sarcopenia in the cirrhotic population was found to be higher (41%) than that (33%) reported in a previous study (60). A higher prevalence was observed in male patients (44%) and those with hepatic encephalopathy (48%). The findings also illustrated a robust correlation between sarcopenia and both the survival rate and mortality in cirrhosis patients. This correlation was substantiated by subgroup analyses based on study location, sample origin, diagnostic criteria, and methodologies. Furthermore, the analysis outcomes indicated a substantial connection between sarcopenia and hepatic encephalopathy in individuals with liver cirrhosis.

A previous systematic reviews and meta-analyses included four articles that assessed the relationship between sarcopenia and cirrhosis. However, one study only pooled retrospective cohorts of patients with sarcopenia and hepatic encephalopathy (61), another study did not include hepatic encephalopathy in the analysis (15), one study solely focused on the prevalence of sarcopenia in patients with cirrhosis (60). A different meta-analysis included a large number of patients after liver transplantation, but all included studies were retrospective observational cohort studies (62). In contrast, our study conducted a more comprehensive and extensive search, additionally analyzed hepatic encephalopathy in patients with liver cirrhosis, and incorporated approximately 50% prospective studies. To ensure the quality of the study, we rigorously controlled the inclusion and exclusion criteria for articles and performed subgroup and sensitivity analyses for all outcomes.

This research revealed that the general prevalence of sarcopenia in individuals with liver cirrhosis stood at 41%, with a notably elevated prevalence among male patients. This finding was in line with previous analyses (15, 60), which could be attributed to the predominance of males in the study samples (67.08%). Furthermore, we established a strong link between sarcopenia and an increased risk of death in cirrhotic patients. Patients with sarcopenia exhibited a higher mortality rate and a 2.13-fold elevated risk of death compared to those without sarcopenia. Sarcopenia posed a significant obstacle to survival. A previous study has demonstrated a higher incidence of complications and a reduced quality of life in patients with sarcopenia (27). The survival rate of patients with sarcopenia was 2.57 times lower than that of patients without sarcopenia. In a subgroup analysis of survival, the pooled HR was higher in patients receiving transjugular intrahepatic portocaval shunts (TIPS) than in the general population (3.71 vs. 2.35). This suggests that TIPS had a more significant impact on survival, and we hypothesized that sarcopenia played a role in worsening survival among cirrhotic patients receiving TIPS. The combined HR was also higher in the cohort containing hepatocellular carcinoma (HCC) patients compared to the cohort without HCC patients (3.38 vs. 2.31), indicating that HCC had a more pronounced effect on survival rates. This highlights the connection between sarcopenia and 5 years cumulative survival in cirrhotic patients.

From a pathophysiologic point of view, sarcopenia results from an imbalance between protein synthesis and degradation caused by multiple pathways. Chronic sarcopenia is characterized by loss of muscle mass, metabolic and biochemical abnormalities, and disruption of protein homeostasis throughout the body (63). Increased hepatic gluconeogenesis in cirrhotic patients is most likely due to limited hepatic glycogen content and insulin resistance, resulting in insufficient supply of branched-chain amino acids and glucose to muscle cells. In addition, recent clinical trials have found testosterone deficiency in cirrhotic patients, indicating increased muscle cell apoptosis and myogenic protein activity (64). Sarcopenia may be also induced by chronic catabolic conditions, such as cachexia due to cirrhosis, increased energy consumption, and decreased food intake due to loss of appetite. Maintaining muscle mass and avoiding rapid loss of muscle mass and transition to sarcopenia appear to be critical for the prognosis of patients with cirrhosis. Therefore, improving survival and quality of life by monitoring body composition and screening for sarcopenia should be a priority in the clinical management of cirrhosis.

Our analysis suggests that sarcopenia constitutes a risk factor for hepatic encephalopathy. In the context of hepatic encephalopathy, ammonia plays a pivotal role in the development of HE in individuals with liver cirrhosis. Previous studies have also demonstrated that the toxicity of ammonia impacts muscles and other organs. In chronic liver disease, muscles play a critical compensatory role in ammonia clearance. However, as the ammonia clearance rate decreases in cirrhotic patients, the compensatory function of patients with sarcopenia weakens. This results in the influx of ammonia into the blood in substantial quantities, greatly increasing the likelihood of hepatic encephalopathy. During hyperammonemia, the loss of muscle mass and subsequent impairment, induced by mitochondrial dysfunction and reduced adenosine triphosphate, or altered protein modification due to various factors, contribute to the onset of sarcopenia, creating a vicious cycle (65). Diagnosing hepatic encephalopathy is vital for the prognosis of patients with liver cirrhosis. However, due to equipment and personnel constraints, hepatic encephalopathy is not routinely examined in clinical practice (66). Therefore, medical staff should be attentive to such patients and implement more targeted screening and interventions for their benefit. The earlier sarcopenia is detected, the sooner the prevention and treatment programs can be initiated to prevent significant impact on HE.

The limitations of this article can be summarized as follows. First, the patient characteristics of the included studies were inconsistent, such as their demographics (i.e., the severity of and causes of cirrhosis) and methods used to determine sarcopenia. This inconsistency may have contributed to the observed heterogeneity. To address this issue, future studies should standardize diagnostic criteria as much as possible to include more patients with cirrhosis and/or sarcopenia with the same characteristics. Second, the majority of the studies included in our analysis were observational cohort studies, potentially introducing bias due to variations in statistical methods, diagnostic criteria, cut-off values for defining sarcopenia, and the distribution of viral liver disease, alcoholic liver disease, and the percentage of cases involving hepatocellular carcinoma (HCC). We attempted to mitigate these limitations through subgroup and sensitivity analyses. Nevertheless, it is imperative to adopt standardized definitions and criteria when conducting meta-analyses of individual sarcopenia data to better elucidate the prevalence of sarcopenia and provide a more comprehensive description of cirrhosis patients with sarcopenia. Finally, we only included studies published in English and may have excluded relevant studies published in other languages, so there may be biases.

In conclusion, this systematic review and meta-analysis establishes that patients with cirrhosis have a prevalence of sarcopenia of 41%, with up to half of these individuals developing cirrhosis due to either alcoholic liver disease or viral hepatitis. Moreover, sarcopenia is closely associated with the survival and mortality rates in patients with cirrhosis, the study reveals that sarcopenia is linked to a greater than twofold rise in the risk of mortality and a decline in survival rates across most subgroups. Hepatic encephalopathy may interact with sarcopenia in patients with cirrhosis. Based on our comprehensive analysis, sarcopenia should be included as an integral component of the initial assessment for all cirrhosis patients.

## Data availability statement

The original contributions presented in the study are included in the article/Supplementary material, further inquiries can be directed to the corresponding author.

## Author contributions

YC: Conceptualization, Formal analysis, Investigation, Methodology, Writing – original draft, Writing – review & editing. MZ: Conceptualization, Formal analysis, Investigation, Writing – original draft, Writing – review & editing. JG: Conceptualization, Formal analysis, Investigation, Writing – review & editing. JJ: Conceptualization, Formal analysis, Investigation, Writing – review & editing. HW: Conceptualization, Supervision, Writing – review & editing. XW: Conceptualization, Supervision, Writing – review & editing.
