# Supplementary material for: Correlation between sarcopenia and cirrhosis: a meta-analysis
Source: Front Nutr. 2024 Jan 10;10:1342100. doi: 10.3389/fnut.2023.1342100 (PMC10805929; doi:10.3389/fnut.2023.1342100)
Supplement: Supplementary file 1 [file Data_Sheet_1.docx]

**Correlation between sarcopenia and cirrhosis: a meta-analysis**

Yifan Cui^1#^, Mingming Zhang^1#^, Jing Guo^1^, Jin Jin^1^, Haijiao Wang^1^, Xinran Wang^1^*

^1^General Surgery Department, Xuanwu Hospital Capital Medical University, Beijing 100053, China

**^#^** Yifan Cui and Mingming Zhang contributed equally to this work.

***Corresponding author:**

Xinran Wang

E-mail: xwsicu2011@163.com

**Table of contents**

**Supplementary Table S1.** Literature search strategy

**Supplementary Figure S1.** Subgroup analyses of HR for survival was performed by source of sample

**Supplementary Figure S2.** Subgroup analyses of HR for survival was performed by country

**Supplementary Figure S3.** Subgroup analyses of HR for survival was performed by hepatocellular carcinoma (HCC)

**Supplementary Figure S4.** Funnel diagram of the study of sarcopenia and the survival rate

**Supplementary Figure S5.** Funnel diagram of the study of sarcopenia and the risk of death

**Supplementary Figure S6.** Subgroup analyses of HR for mortality was performed by country

**Supplementary Figure S7.** Subgroup analyses of HR for mortality was performed by definition of sarcopenia

**Supplementary Figure S8.** Subgroup analyses of HR for mortality was performed by hepatocellular carcinoma (HCC)

**Supplementary Figure S9.** Subgroup analyses of HR for mortality was performed by hepatocellular carcinoma (HCC)

**Supplementary Table S1.** Literature search strategy

| Search number | Query | Results |
| --- | --- | --- |
| **1.Pubmed** | |  |
| #1 | sarcopenia[MeSH Terms] | 9,284 |
| #2 | sarcopenia[Title/Abstract] OR sarcopenias[Title/Abstract] OR muscular atrophy[Title/Abstract] OR muscle weakness[Title/Abstract] OR muscular weakness[Title/Abstract] OR Skeletal muscle depletion[Title/Abstract] OR Frail*[Title/Abstract] OR Cachexia[Title/Abstract] OR muscle strength[Title/Abstract] OR Muscle function[Title/Abstract] OR Muscle mass[Title/Abstract] OR lean body weight[Title/Abstract] | 137,214 |
| #3 | (sarcopenia[MeSH Terms]) OR (sarcopenia[Title/Abstract] OR sarcopenias[Title/Abstract] OR muscular atrophy[Title/Abstract] OR muscle weakness[Title/Abstract] OR muscular weakness[Title/Abstract] OR Skeletal muscle depletion[Title/Abstract] OR Frail*[Title/Abstract] OR Cachexia[Title/Abstract] OR muscle strength[Title/Abstract] OR Muscle function[Title/Abstract] OR Muscle mass[Title/Abstract] OR lean body weight[Title/Abstract]) | 137,670 |
| #4 | Liver Cirrhosis[MeSH Terms] | 102,097 |
| #5 | Hepatic Cirrhosis[Title/Abstract] OR Liver Fibrosis[Title/Abstract] OR Hepatic Fibrosis[Title/Abstract] OR Cirrhosis[Title/Abstract] OR 'liver cirrhosis, alcoholic'[Title/Abstract] OR 'liver cirrhosis, biliary'[Title/Abstract] OR fatty liver[Title/Abstract] | 164,463 |
| #6 | (Liver Cirrhosis[MeSH Terms]) OR (Hepatic Cirrhosis[Title/Abstract] OR Liver Fibrosis[Title/Abstract] OR Hepatic Fibrosis[Title/Abstract] OR Cirrhosis[Title/Abstract] OR 'liver cirrhosis, alcoholic'[Title/Abstract] OR 'liver cirrhosis, biliary'[Title/Abstract] OR fatty liver[Title/Abstract]) | 192,012 |
| #7 | hepatic encephalopathy[MeSH Terms] | 11,206 |
| #8 | hepatic encephalopathy[Title/Abstract] OR Hepatic Encephalopathies[Title/Abstract] OR Portal-Systemic Encephalopathy[Title/Abstract] OR Portal-Systemic Encephalopathies[Title/Abstract] OR Hepatocerebral Encephalopathy[Title/Abstract] OR Hepatocerebral Encephalopathies[Title/Abstract] OR Portosystemic Encephalopathy[Title/Abstract] OR Portosystemic Encephalopathies[Title/Abstract] OR Hepatic Coma*[Title/Abstract] OR Hepatic Stupor*[Title/Abstract] OR ammoniac encephalopathy[Title/Abstract] OR hepato cerebral disease[Title/Abstract] OR hepatocerebral disease[Title/Abstract] OR hepatocerebral syndrome[Title/Abstract] OR hepatoencephalopathy[Title/Abstract] OR hepatogenous encephalopathy[Title/Abstract] OR liver encephalopathy[Title/Abstract] OR porta cava encephalopathy[Title/Abstract] OR portal encephalopathy[Title/Abstract] OR portocaval encephalopathy[Title/Abstract] OR Fulminant Hepatic Failure Cerebral Edema[Title/Abstract] | 12,218 |
| #9 | (hepatic encephalopathy[MeSH Terms]) OR (hepatic encephalopathy[Title/Abstract] OR Hepatic Encephalopathies[Title/Abstract] OR Portal-Systemic Encephalopathy[Title/Abstract] OR Portal-Systemic Encephalopathies[Title/Abstract] OR Hepatocerebral Encephalopathy[Title/Abstract] OR Hepatocerebral Encephalopathies[Title/Abstract] OR Portosystemic Encephalopathy[Title/Abstract] OR Portosystemic Encephalopathies[Title/Abstract] OR Hepatic Coma*[Title/Abstract] OR Hepatic Stupor*[Title/Abstract] OR ammoniac encephalopathy[Title/Abstract] OR hepato cerebral disease[Title/Abstract] OR hepatocerebral disease[Title/Abstract] OR hepatocerebral syndrome[Title/Abstract] OR hepatoencephalopathy[Title/Abstract] OR hepatogenous encephalopathy[Title/Abstract] OR liver encephalopathy[Title/Abstract] OR porta cava encephalopathy[Title/Abstract] OR portal encephalopathy[Title/Abstract] OR portocaval encephalopathy[Title/Abstract] OR Fulminant Hepatic Failure Cerebral Edema[Title/Abstract]) | 16,532 |
| #10 | ((Liver Cirrhosis[MeSH Terms]) OR (Hepatic Cirrhosis[Title/Abstract] OR Liver Fibrosis[Title/Abstract] OR Hepatic Fibrosis[Title/Abstract] OR Cirrhosis[Title/Abstract] OR 'liver cirrhosis, alcoholic'[Title/Abstract] OR 'liver cirrhosis, biliary'[Title/Abstract] OR fatty liver[Title/Abstract])) OR ((hepatic encephalopathy[MeSH Terms]) OR (hepatic encephalopathy[Title/Abstract] OR Hepatic Encephalopathies[Title/Abstract] OR Portal-Systemic Encephalopathy[Title/Abstract] OR Portal-Systemic Encephalopathies[Title/Abstract] OR Hepatocerebral Encephalopathy[Title/Abstract] OR Hepatocerebral Encephalopathies[Title/Abstract] OR Portosystemic Encephalopathy[Title/Abstract] OR Portosystemic Encephalopathies[Title/Abstract] OR Hepatic Coma*[Title/Abstract] OR Hepatic Stupor*[Title/Abstract] OR ammoniac encephalopathy[Title/Abstract] OR hepato cerebral disease[Title/Abstract] OR hepatocerebral disease[Title/Abstract] OR hepatocerebral syndrome[Title/Abstract] OR hepatoencephalopathy[Title/Abstract] OR hepatogenous encephalopathy[Title/Abstract] OR liver encephalopathy[Title/Abstract] OR porta cava encephalopathy[Title/Abstract] OR portal encephalopathy[Title/Abstract] OR portocaval encephalopathy[Title/Abstract] OR Fulminant Hepatic Failure Cerebral Edema[Title/Abstract])) | 202,261 |
| #11 | ((sarcopenia[MeSH Terms]) OR (sarcopenia[Title/Abstract] OR sarcopenias[Title/Abstract] OR muscular atrophy[Title/Abstract] OR muscle weakness[Title/Abstract] OR muscular weakness[Title/Abstract] OR Skeletal muscle depletion[Title/Abstract] OR Frail*[Title/Abstract] OR Cachexia[Title/Abstract] OR muscle strength[Title/Abstract] OR Muscle function[Title/Abstract] OR Muscle mass[Title/Abstract] OR lean body weight[Title/Abstract])) AND (((Liver Cirrhosis[MeSH Terms]) OR (Hepatic Cirrhosis[Title/Abstract] OR Liver Fibrosis[Title/Abstract] OR Hepatic Fibrosis[Title/Abstract] OR Cirrhosis[Title/Abstract] OR 'liver cirrhosis, alcoholic'[Title/Abstract] OR 'liver cirrhosis, biliary'[Title/Abstract] OR fatty liver[Title/Abstract])) OR ((hepatic encephalopathy[MeSH Terms]) OR (hepatic encephalopathy[Title/Abstract] OR Hepatic Encephalopathies[Title/Abstract] OR Portal-Systemic Encephalopathy[Title/Abstract] OR Portal-Systemic Encephalopathies[Title/Abstract] OR Hepatocerebral Encephalopathy[Title/Abstract] OR Hepatocerebral Encephalopathies[Title/Abstract] OR Portosystemic Encephalopathy[Title/Abstract] OR Portosystemic Encephalopathies[Title/Abstract] OR Hepatic Coma*[Title/Abstract] OR Hepatic Stupor*[Title/Abstract] OR ammoniac encephalopathy[Title/Abstract] OR hepato cerebral disease[Title/Abstract] OR hepatocerebral disease[Title/Abstract] OR hepatocerebral syndrome[Title/Abstract] OR hepatoencephalopathy[Title/Abstract] OR hepatogenous encephalopathy[Title/Abstract] OR liver encephalopathy[Title/Abstract] OR porta cava encephalopathy[Title/Abstract] OR portal encephalopathy[Title/Abstract] OR portocaval encephalopathy[Title/Abstract] OR Fulminant Hepatic Failure Cerebral Edema[Title/Abstract]))) | 1,612 |
| **2.Cochrane** | |  |
| #1 | (sarcopenia):ti,ab,kw OR (sarcopenias):ti,ab,kw OR (muscular atrophy):ti,ab,kw OR (muscle weakness):ti,ab,kw OR (muscular weakness):ti,ab,kw (Word variations have been searched) | 8444 |
| #2 | (Skeletal muscle depletion):ti,ab,kw OR (Frail*):ti,ab,kw OR (Cachexia):ti,ab,kw OR (muscle strength):ti,ab,kw OR (Muscle function):ti,ab,kw (Word variations have been searched) | 53509 |
| #3 | (Muscle mass):ti,ab,kw OR (lean body weight):ti,ab,kw (Word variations have been searched) | 16365 |
| #4 | #1 OR #2 OR #3 | 63654 |
| #5 | (Liver Cirrhosis):ti,ab,kw OR (Hepatic Cirrhosis):ti,ab,kw OR (Liver Fibrosis):ti,ab,kw OR (Hepatic Fibrosis):ti,ab,kw OR (Cirrhosis):ti,ab,kw (Word variations have been searched) | 13659 |
| #6 | (liver cirrhosis, alcoholic):ti,ab,kw OR (liver cirrhosis, biliary):ti,ab,kw OR (fatty liver):ti,ab,kw (Word variations have been searched) | 8167 |
| #7 | (hepatic encephalopathy):ti,ab,kw OR (Hepatic Encephalopathies):ti,ab,kw OR (Portal-Systemic Encephalopathy):ti,ab,kw OR (Portal-Systemic Encephalopathies):ti,ab,kw OR (Hepatocerebral Encephalopathy):ti,ab,kw (Word variations have been searched) | 2150 |
| #8 | (Hepatocerebral Encephalopathies):ti,ab,kw OR (Portosystemic Encephalopathy):ti,ab,kw OR (Portosystemic Encephalopathies):ti,ab,kw OR (Hepatic Coma*):ti,ab,kw OR (Hepatic Stupor*):ti,ab,kw (Word variations have been searched) | 387 |
| #9 | (Fulminant Hepatic Failure with Cerebral Edema):ti,ab,kw OR (ammoniac encephalopathy):ti,ab,kw OR (hepato cerebral disease):ti,ab,kw OR (hepatocerebral disease):ti,ab,kw OR (hepatocerebral syndrome):ti,ab,kw (Word variations have been searched) | 16 |
| #10 | (hepatoencephalopathy):ti,ab,kw OR (hepatogenous encephalopathy):ti,ab,kw OR (liver encephalopathy):ti,ab,kw OR (porta cava encephalopathy):ti,ab,kw OR (portal encephalopathy):ti,ab,kw (Word variations have been searched) | 1929 |
| #11 | (portocaval encephalopathy):ti,ab,kw | 19 |
| #12 | #5 OR #6 | 18407 |
| #13 | #7 OR #8 OR #9 OR #10 OR #11 | 2486 |
| #14 | #12 OR #13 | 19432 |
| #15 | #4 AND #14 | 682 |
| **3.Embase** | |  |
| #1 | 'sarcopenia'/exp | 19986 |
| #2 | sarcopenia:ab,ti OR sarcopenias:ab,ti OR 'muscular atrophy':ab,ti OR 'muscle weakness':ab,ti OR 'muscular weakness':ab,ti OR 'skeletal muscle depletion':ab,ti OR frail*:ab,ti OR cachexia:ab,ti OR 'muscle strength':ab,ti OR 'muscle function':ab,ti OR 'muscle mass':ab,ti OR 'lean body weight':ab,ti | 195425 |
| #3 | #1 OR #2 | 197853 |
| #4 | 'liver cirrhosis':ab,ti OR 'hepatic cirrhosis':ab,ti OR 'liver fibrosis':ab,ti OR 'hepatic fibrosis':ab,ti OR cirrhosis:ab,ti OR 'liver cirrhosis, alcoholic':ab,ti OR 'liver cirrhosis, biliary':ab,ti OR 'fatty liver':ab,ti | 249148 |
| #5 | 'liver cirrhosis'/exp | 205080 |
| #6 | #4 OR #5 | 312563 |
| #7 | 'hepatic encephalopathy'/exp | 25090 |
| #8 | 'hepatic encephalopathy':ab,ti OR 'hepatic encephalopathies':ab,ti OR 'portal-systemic encephalopathy':ab,ti OR 'portal-systemic encephalopathies':ab,ti OR 'hepatocerebral encephalopathy':ab,ti OR 'hepatocerebral encephalopathies':ab,ti OR 'portosystemic encephalopathy':ab,ti OR 'portosystemic encephalopathies':ab,ti OR 'hepatic coma*':ab,ti OR 'hepatic stupor*':ab,ti OR 'fulminant hepatic failure with cerebral edema':ab,ti OR 'ammoniac encephalopathy':ab,ti OR 'hepato cerebral disease':ab,ti OR 'hepatocerebral disease':ab,ti OR 'hepatocerebral syndrome':ab,ti OR hepatoencephalopathy:ab,ti OR 'hepatogenous encephalopathy':ab,ti OR 'liver encephalopathy':ab,ti OR 'porta cava encephalopathy':ab,ti OR 'portal encephalopathy':ab,ti OR 'portocaval encephalopathy':ab,ti | 19602 |
| #9 | #7 OR #8 | 28948 |
| #10 | #6 OR #9 | 327351 |
| #11 | #3 AND #10 | 3313 |
| **4.Web of science** | |  |
| #1 | "(((((((((((TS=(sarcopenia)) OR TS=(sarcopenias)) OR TS=( muscular atrophy)) OR TS=(muscle weakness)) OR TS=(muscular weakness)) OR TS=(Skeletal muscle depletion)) OR TS=(Frail*)) OR S=(Cachexia)) OR TS=(muscle strength)) OR TS=(Muscle function)) OR TS=(Muscle mass)) OR TS=(lean body weight) | 723176 |
| #2 | (((((((TS=(Liver Cirrhosis)) OR TS=(Hepatic Cirrhosis)) OR TS=(Liver Fibrosis)) OR TS=(Hepatic Fibrosis)) OR TS=(Cirrhosis)) OR TS=(liver cirrhosis, alcoholic)) OR TS=(liver cirrhosis, biliary)) OR TS=(fatty liver) | 435866 |
| #3 | "((((((((((((((((((((TS=(hepatic encephalopathy)) OR TS=(Hepatic Encephalopathies)) OR TS=(Portal-Systemic Encephalopathy)) OR TS=(Portal-Systemic Encephalopathies)) OR TS=(Hepatocerebral Encephalopathy)) OR TS=(Hepatocerebral Encephalopathies)) OR TS=(Portosystemic Encephalopathy)) OR TS=(Portosystemic Encephalopathies)) OR TS=(Hepatic Coma*)) OR TS=(Hepatic Stupor*)) OR TS=(Fulminant Hepatic Failure with Cerebral Edema)) OR TS=(ammoniac encephalopathy)) OR TS=(hepato cerebral disease)) OR TS=(hepatocerebral disease)) OR TS=(hepatocerebral syndrome)) OR TS=(hepatoencephalopathy)) OR TS=(hepatogenous encephalopathy)) OR TS=(liver encephalopathy)) OR TS=(porta cava encephalopathy)) OR TS=(portal encephalopathy)) OR TS=(portocaval encephalopathy) | 35362 |
| #4 | #2 OR #3 | 456680 |
| #5 | #4 AND #1 | 14464 |

**
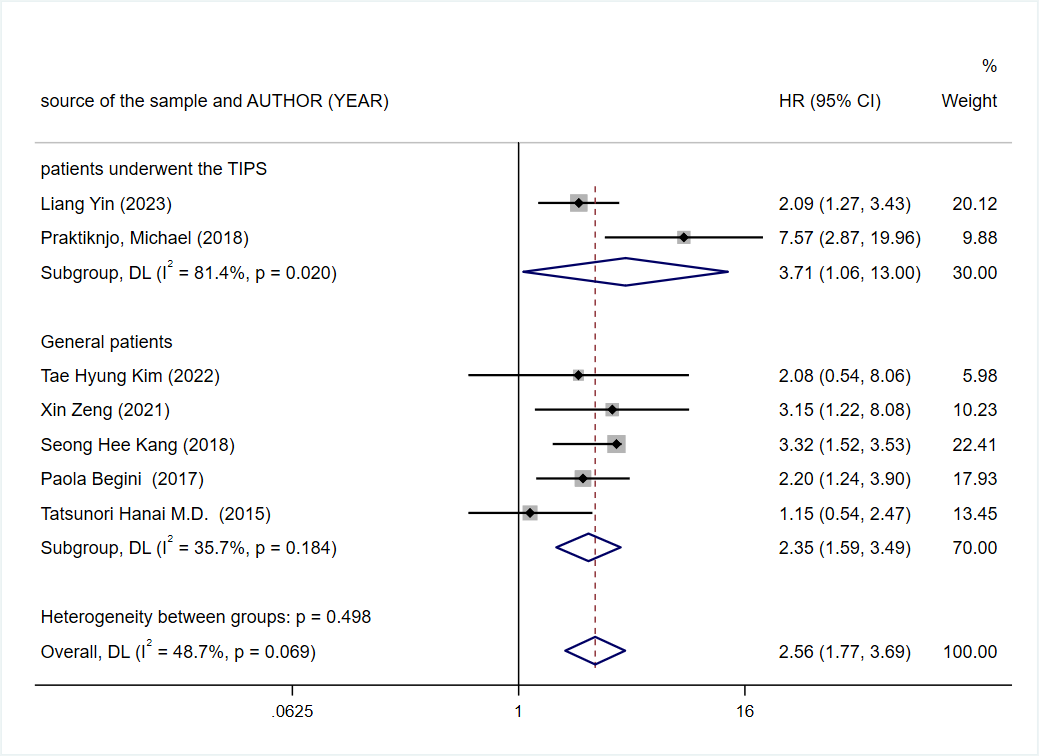
**

**Supplementary Figure S1.** Subgroup analyses of HR for survival was performed by source of sample


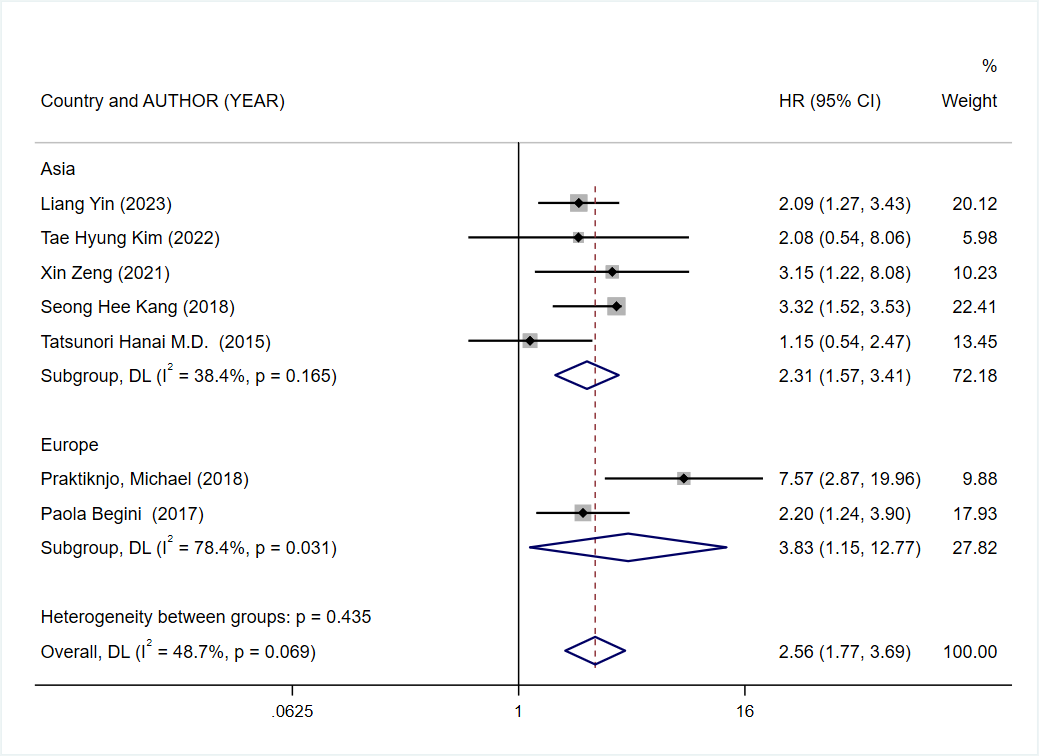


**Supplementary Figure S2.** Subgroup analyses of HR for survival was performed by country

**
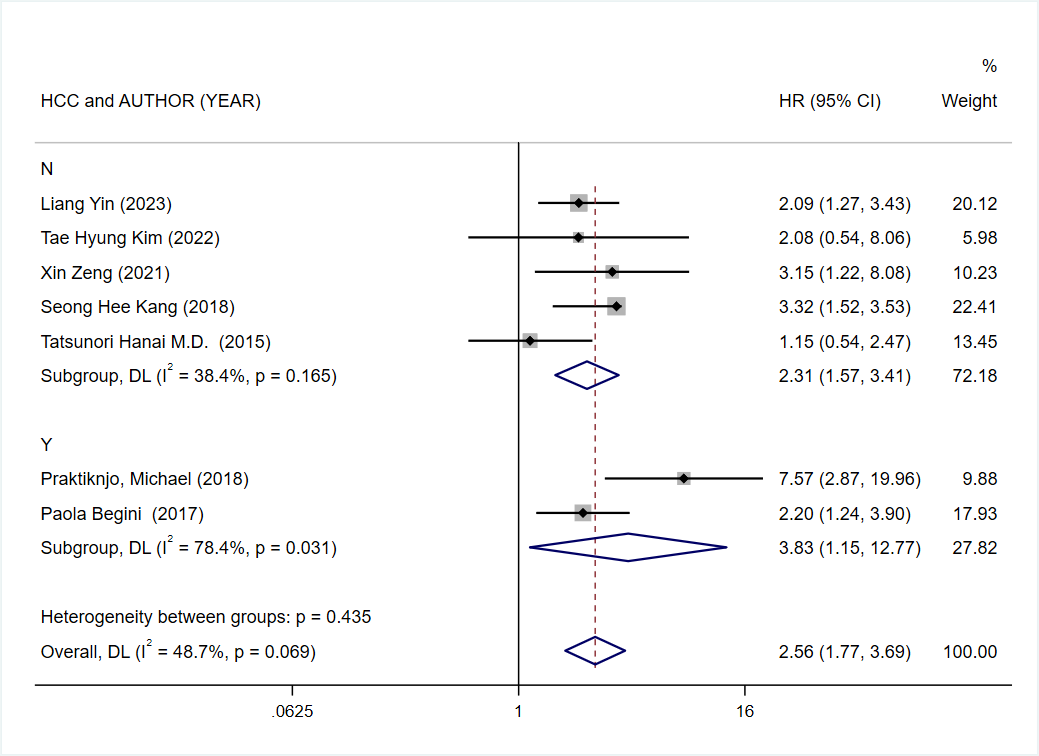
**

**Supplementary Figure S3.** Subgroup analyses of HR for survival was performed by hepatocellular carcinoma (HCC)

**
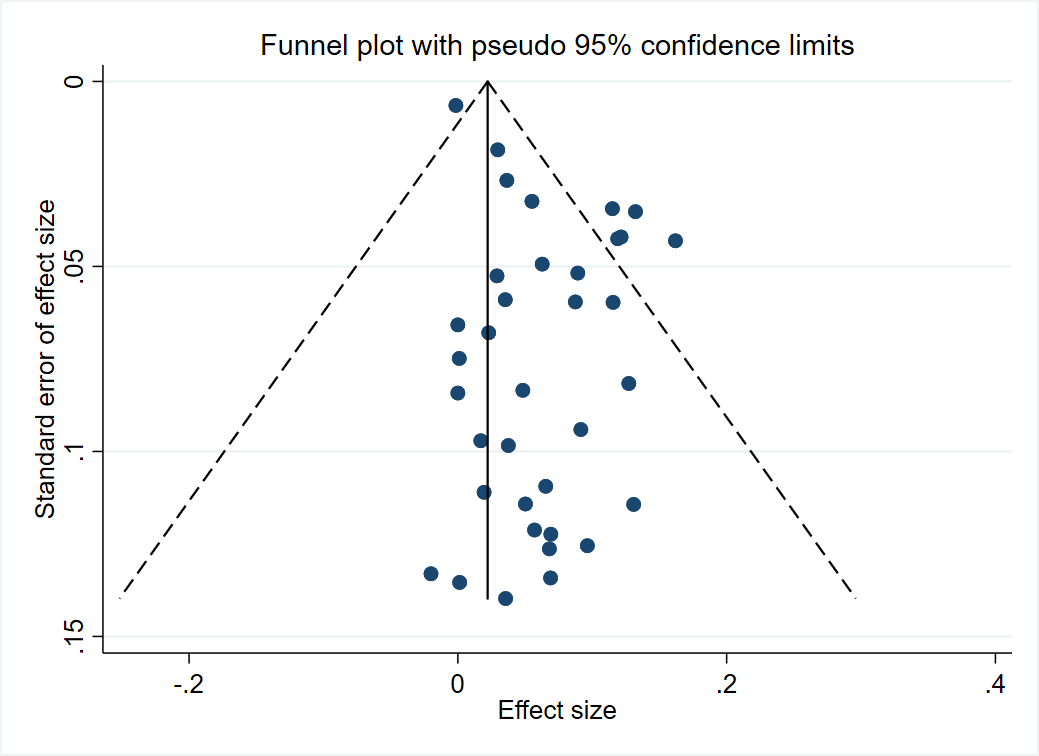
Supplementary Figure S4.** Funnel diagram of the study of sarcopenia and the survival rate

**
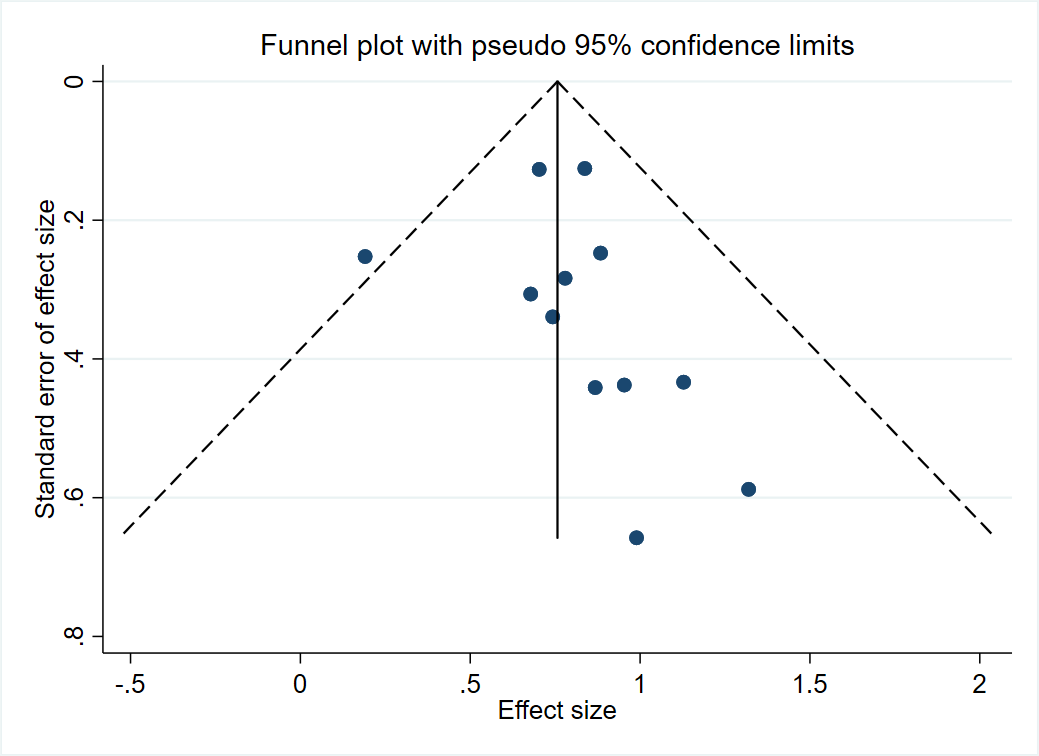
Supplementary Figure S5.** Funnel diagram of the study of sarcopenia and the risk of death

**
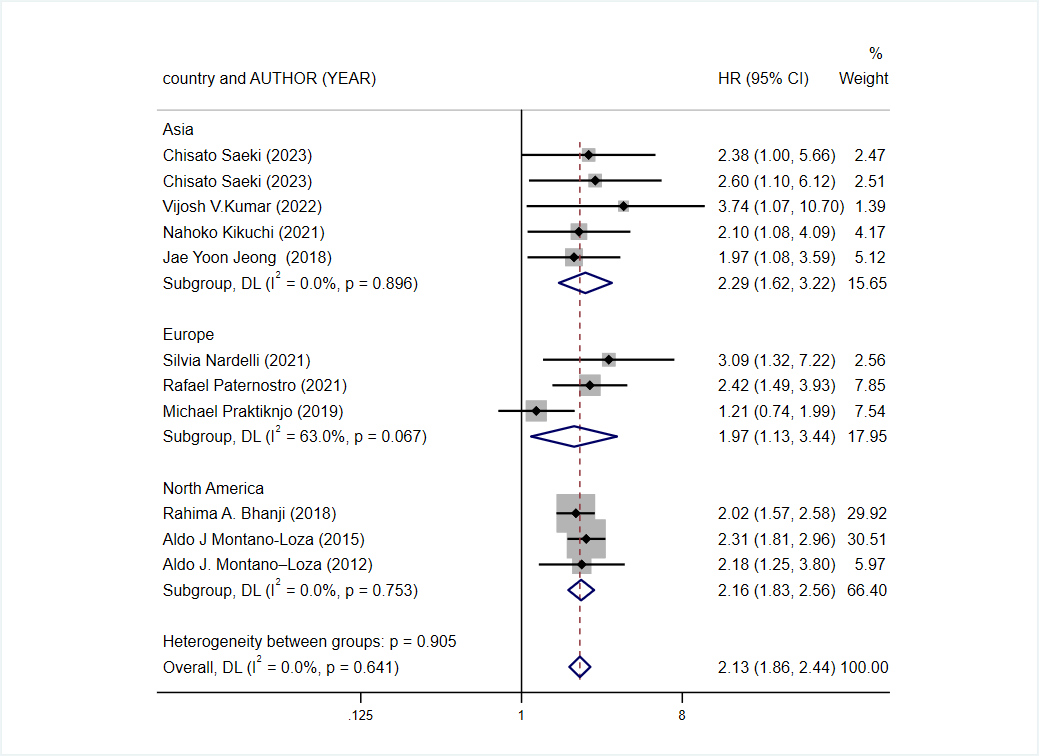
Supplementary Figure S6.** Subgroup analyses of HR for mortality was performed by country

**
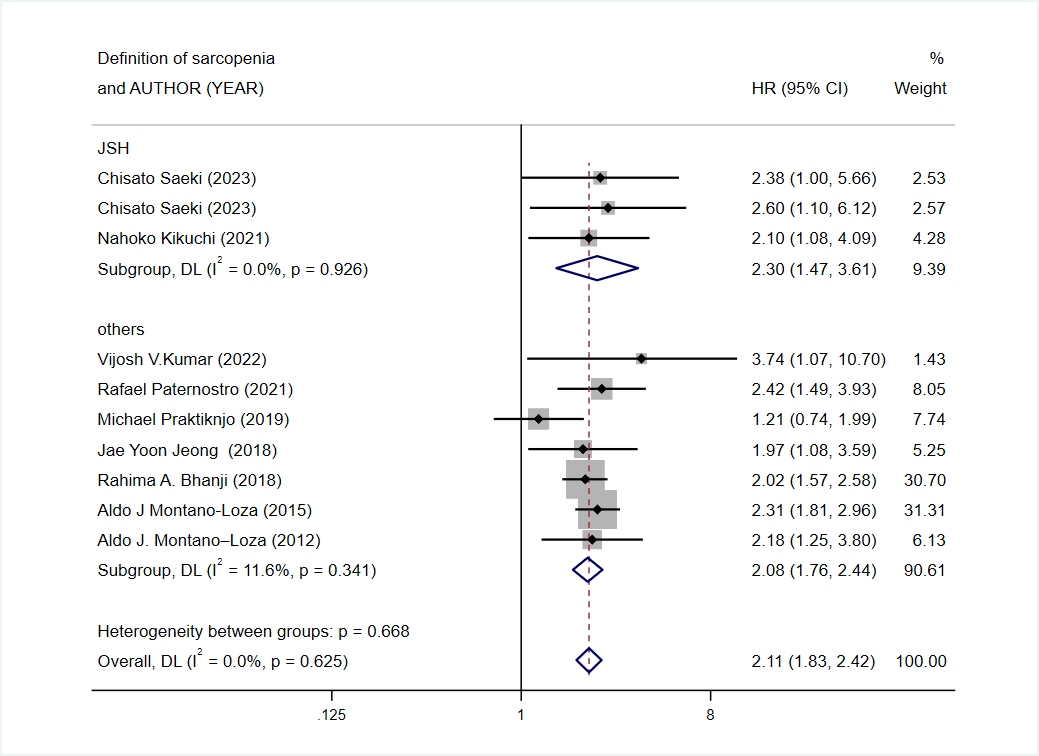
Supplementary Figure S7.** Subgroup analyses of HR for mortality was performed by definition of sarcopenia

**
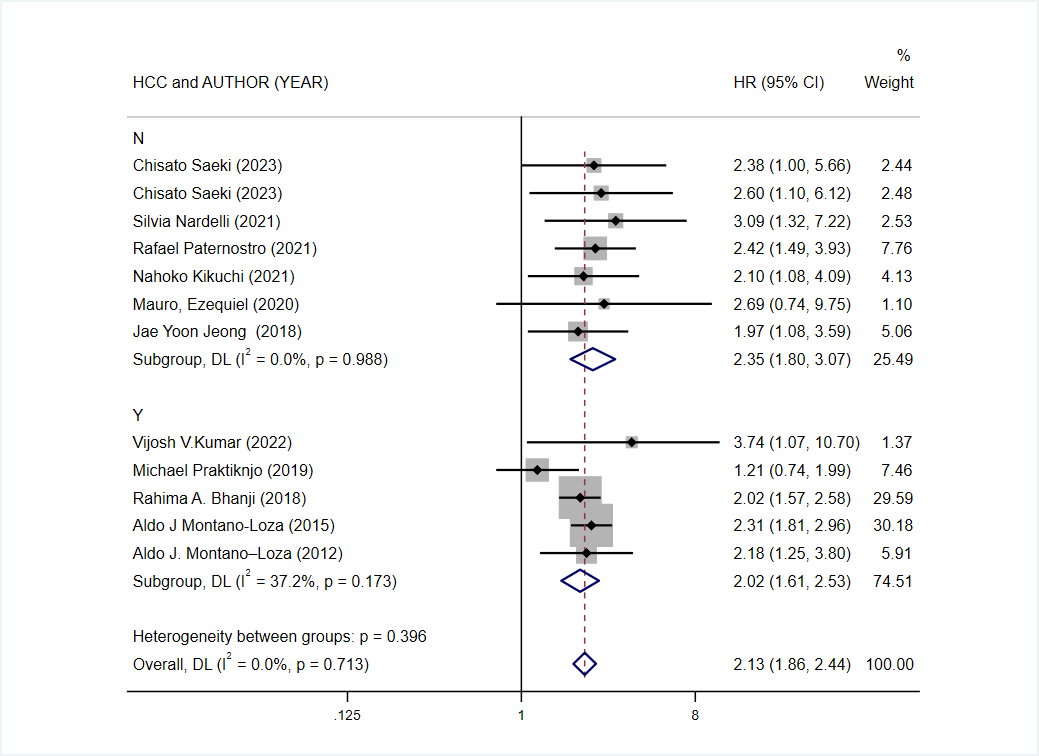
Supplementary Figure S8.** Subgroup analyses of HR for mortality was performed by hepatocellular carcinoma (HCC)


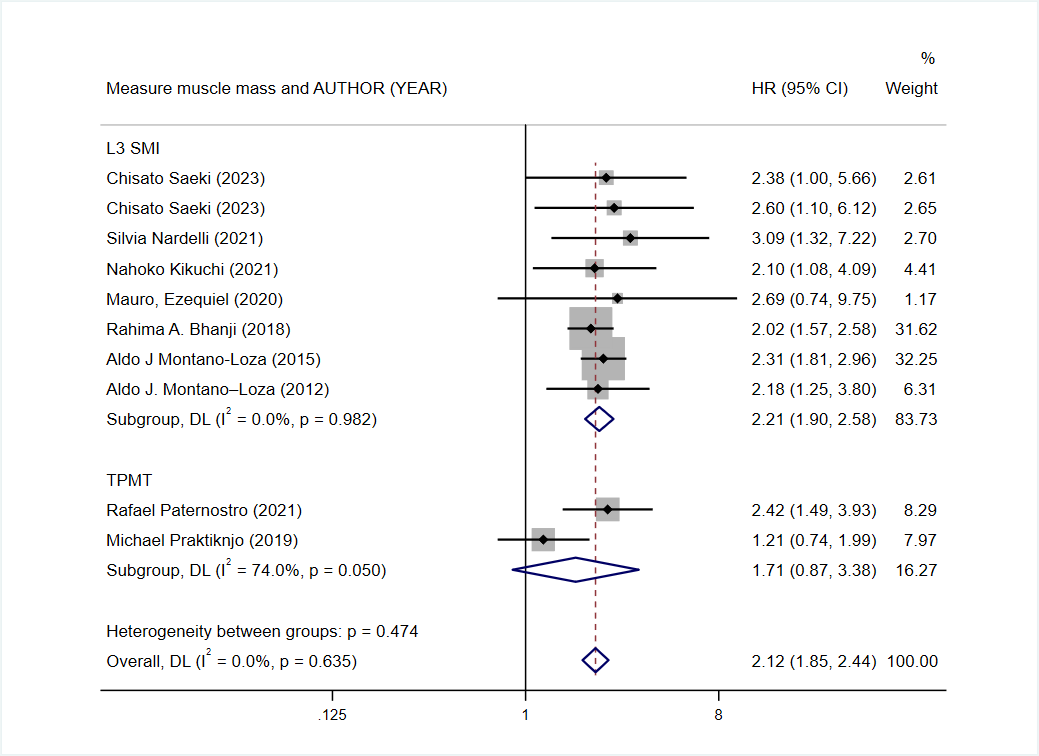


**Supplementary Figure S9.** Subgroup analyses of HR for mortality was performed by measure muscle mass
